# Anaesthesia for chest wall reconstruction in a patient with Poland syndrome: CARE-compliant case report and literature review

**DOI:** 10.1186/s12871-018-0518-4

**Published:** 2018-05-24

**Authors:** Lingli Gui, Shiqian Shen, Wei Mei

**Affiliations:** 1 Department of Anesthesiology and Pain medicine, Tongji Hospital, Tongji Medical College, Huazhong University of Science and Technology, 1095 Jiefang Road, Wuhan, 430030 People’s Republic of China; 2 Department of Anesthesia, Critical Care and Pain Medicine, Massachsetts General Hospital, Harvard Medical School, 55 Fruit St, Boston, MA 02129 USA

**Keywords:** Poland syndrome, Anesthesia, Thoracic wall

## Abstract

**Background:**

Poland syndrome is a rare congenital disease, characterized by agenesis/hypoplasia of the pectoralis major muscle, usually associated with variable thoracic anomalies that needed chest wall reconstruction under general anesthesia. Anaesthetic management in Poland syndrome has scarcely been described.

**Case presentation:**

Here, we present our anaesthetic management of Nuss procedure for chest wall correction in a 5 years old patient with Poland syndrome. We also reviewed the reports of anaesthetic management of Poland syndrome by searching Pubmed, and summarize the perioperative procedures that may warrant a safe surgery.

**Conclusions:**

Examinations before surgery, intraoperative monitoring, choice of general anesthetics and pain management after surgery should all be contemplated.

## Background

Poland syndrome (PS) is characterized by hypoplasia / agenesis of the pectoralis major muscle, variably associated with upper limb anomalies (ULA)and/or thoracic anomalies (TA) [1, 2]. A wide range of TA is involved in PS, which includes thoracic skelecton anomalies, such as chest wall depression, rib hypoplasia to aplasia, sternal anomalies, as well as thoracic soft tissue anomalies, such as subtle breast, limited subcutaneous fat and axillary hair absence. ULA can be as severe as phocomelia-like deficiency or not present, and be classic with syndactyly and variable degrees of brachydactyly. Except for what mentioned above, dextrocardia, renal malformations and vertebral anomalies have been reported in rare PS cases [3–5]. This disease has an estimated incidence of 1:30,000 to 1:80,000 of live births [6]. The condition is more frequent among males (female to male ratio is 1:2 to 1:3), and more commonly seen in the right side of the thorax as opposed to the left (3:2) [7].

The patients usually need surgery to correct chest wall deformities and/or syndactyly during childhood, and to correct pectoral and breast deformities as adults, which need anaesthesia. Few cases of patients with PS have been reported, and anaesthetic management in PS has scarcely been described in detail. We present here our anaesthetic management of thoracoscopy assisted minimally invasive repair of pectus excavatum (MIRPE), which is also called Nuss procedure that is named after the author who reported the technique first, for chest wall correction in a patient with Poland syndrome. We also reviewed the reports of anaesthetic management of Poland syndrome by searching Pubmed, and summarize the perioperative procedures that may warrant a safe surgery. The reporting of this case was approved by the Institutional Review Board and the patient’s parents gave written informed consent for the report and publication of their clinical details and images.

## Case presentation

**Fig. 1:**
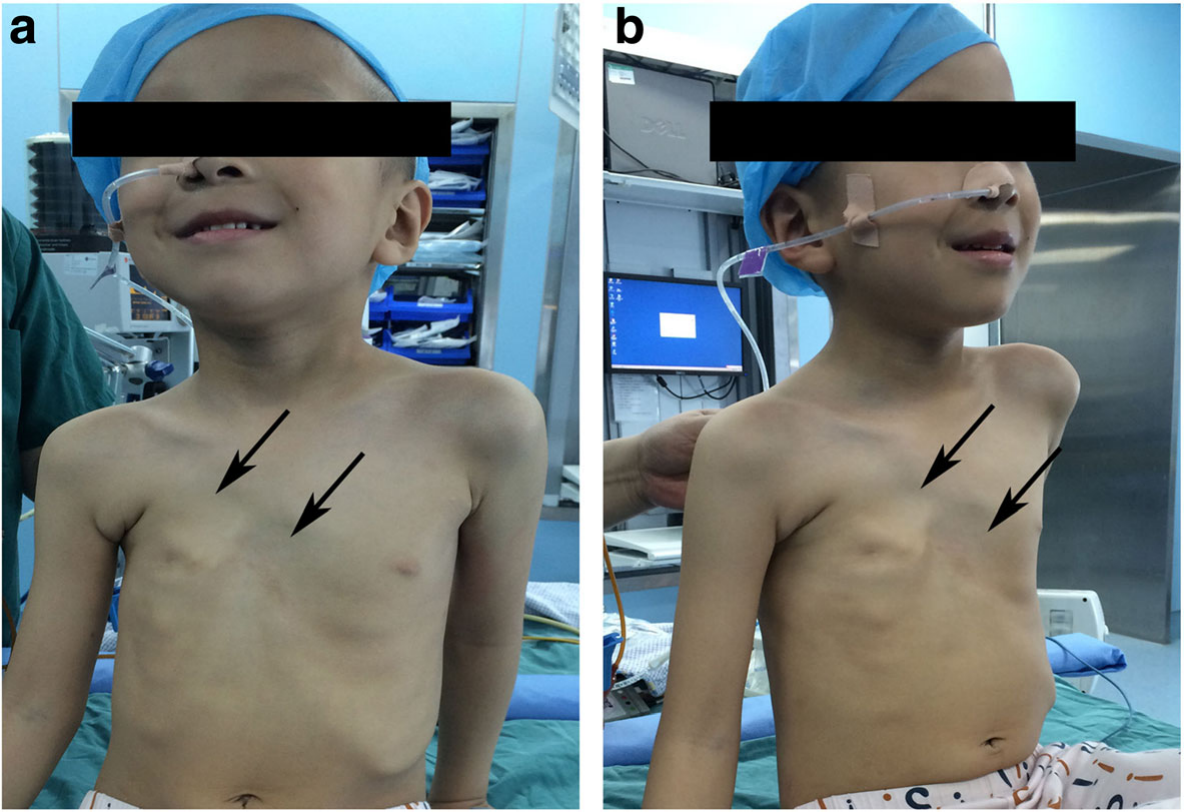
Photographs of the patient showing the right thoracic deformity. Positive (**a**) and side (**b**) photographs show the smaller right thoracic cage, the depressed sternum (arrowheads), the 3rd rib cartilage defect (arrowheads) and the right anterior chest wall musculature loss

**Fig. 2:**
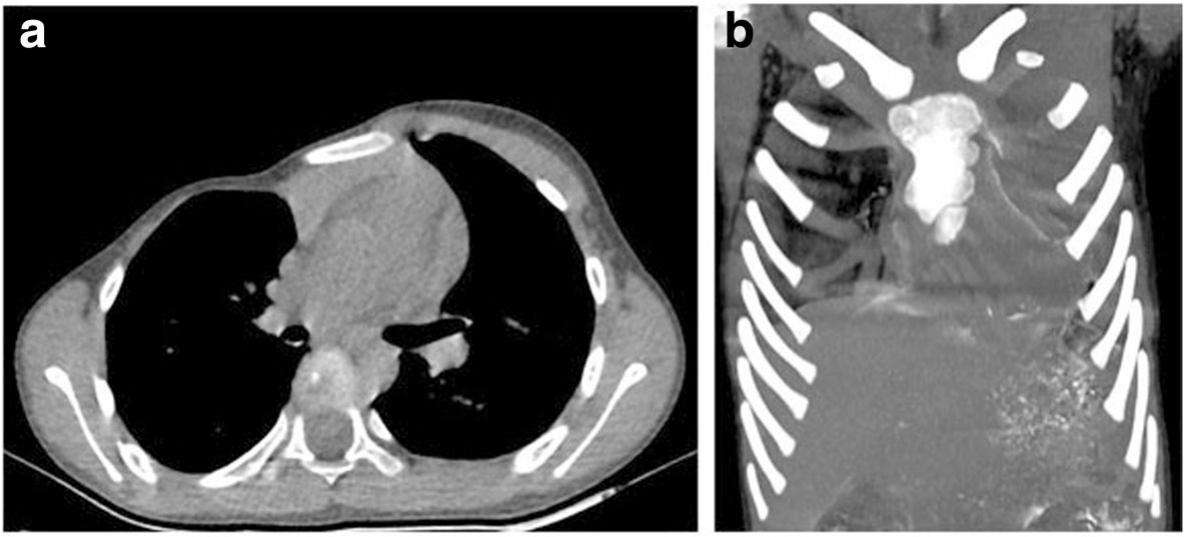
High resolution computed tomography of three-dimensional reconstruction of chest. **a** Depression of the sternum which was rotated to the right and absence of the right chest wall soft tissue. **b** Multiple ribs abnormally extended on right side, particularly from the right 3rd to 7th rib, parasternal absence of the right partial 3rd rib cartilage(arrowheads)

**Fig. 3:**
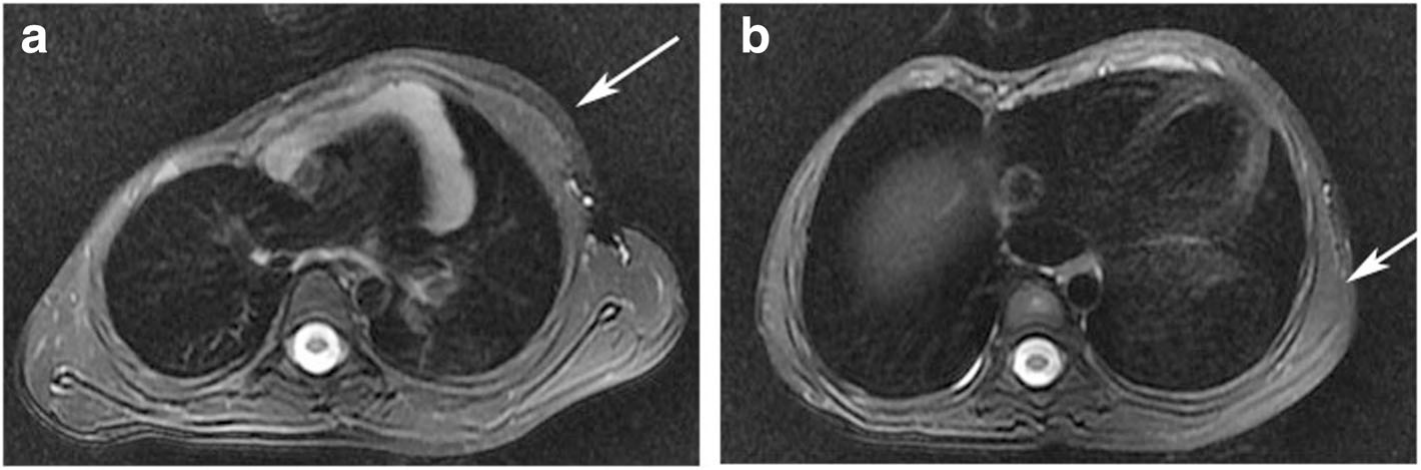
Chest magnetic resonance imaging. It revealed the pectoralis major and minor(**a**, arrowheads) and latissimus dorsi muscles(**b**, arrowheads) of the left side, and the absence of these muscles of the right side

A 5-year and seven-month-old, 23-kg boy, 1.18 m tall, BMI 16.16, was admitted to the hospital suffering from congenital chest wall defect. Preoperative examination revealed deformity of the right thoracic cage. His sternum was depressed at its lower half section, and made a rightward rotation, which showed a profound depression of the right anterior chest wall. The patient had a sternocostal cartilage defect of approximately 4 × 4 cm^2^at the right side of the middle section of the sternum, and right anterior chest wall musculature loss. Paradoxical respiratory movements could be seen upon deep inhalation and pulsations of the heart were visible through the defect (Fig. 1a, b). Decreased excursion of the right hemithorax was revealed by palpation of the thorax, and decreased breath sounds was detected by auscultation on the right. Heart auscultation was normal. His right nipple was hypoplastic, and slightly inferior to the left nipple. There was no associated syndactyly or brachydactyly. The patient was asymptomatic. The chest X-ray showed a smaller right thoracic cage and an elevated right hemidiaphragm. High resolution computed tomography of three-dimensional reconstruction revealed a depression of the sternum, which was rotated to right, multiple ribs abnormally extended on right side, particularly from the right 3rd to 7th rib, parasternal absence of the right partial 3rd costal rib cartilage and absence of the right chest wall soft tissue, (Fig. 2a, b). Moreover, chest magnetic resonance imaging detected the absence of the pectoralis major, pectoralis minor, and latissimus dorsi muscles (Fig. 3a, b). Bilateral subclavian and brachial artery color Doppler echocardiography were normal and showed no angiostenosis on either side. Laboratory findings were normal. No abnormality was found on examination of the other systems. Chromosomal analysis of the patient has the normal testing result. The patient was born of an uneventful pregnancy and delivery. The patient’s elder sister showed no sign of deformity. The family history was unremarkable. The patient had no past history of anesthesia or surgery. He was diagnosed with Poland syndrome. Thoracoscopy assisted Nuss procedure was planned to correct the right chest deformity.

The patient was comfortable at rest and cheerful. Prior to surgery the patient fasted for 8 hours. Intravenous line was placed in the in-patient department. Heart rate was 129 bpm via ECG, respiratory rate was 22 per minute and pulse oximetry saturation was 100% in room air when the patient arrived in the operation room. Noninvasive blood pressure (NIBP) measured at left and right arms were 111/66 mmHg and 110/65 mmHg, respectively. The left upper arm was used to measure NIBP to avoid perturbation of blood pressure measurement. Airway examination did not show any anticipated difficulty. Midazolam (0.02 mg.kg^− 1^) was given intravenously as premedication. General anesthesia with controlled ventilation was planned. Anesthesia induction was performed with 4 mg.kg^− 1^of propofol after 0.3 μg.kg^− 1^ of sufentanil and tracheal intubation with an ID 5.0 mm cuffed tube was facilitated with 0.6 mg.kg^− 1^ of rocuronium. Propfol (8-10 mg.kg^− 1^.h^− 1^) and remifentanil (0.2 μg.kg^− 1^.min^− 1^) were used to maintain anesthesia. After intubation, pressure controlled ventilation was given (P 15cmH_2_O, R 16 bpm, I:E 1:2), end-tidal CO_2_ (EtCO_2_) was maintained at about 37 mmHg. Nasopharyngeal temperature was monitored at 37.3 °C, patient warming system (warm touch™ 5200) and warm fluid via intravenous line were used to keep the patient warm. Bilateral single-shot thoracic paravertebral nerve bloke was given using 0.2% ropivocaine 10 ml per lateral at the level betweenT4-T5 under ultrasound guidance. After thoracic paravertebral block, the patient’s heart rate slowly dropped to 60 bpm and responded to 0.01 mg.kg^− 1^ atropine. Small incisions were made in the two symmetrical axillary midline areas of the chest along the lowest funnel point, at theT4-T5 intercostal level. Subcutaneous tunnels were made from the incisions to the funnel crest. Guided by thoracoscopy, an introducer was advanced through the right funnel crest to the sternum. A severe ST segment depression and a massive drop of EtCO_2_ (from 34 mmHg to 28 mmHg) and heart rate (from 110 bpm to 40 bpm) were evident. The introducer was removed immediately, ECG, EtCO_2_ and heart rate all spontaneously recovered. No injury to the heart and great vessels were revealed by thoracoscopy. Compression on heart or great vessels by the introducer was suspected responsible for the transient cardiopulmonary collapse. After successful placement of an artery line in left hand and a center vein catheter at right neck, surgery recommenced. The patient was placed in Trendleberg’s position to warrant good filling of the heart. Bolus of saline (20 ml saline), atropine (0.02 mg.kg^− 1^) and epinephrine (0.3 μg.kg^− 1^) were given intermediately to keep arterial blood pressure (ABP) above 60/40 mmHg and HR above 100 bpm. During the placement of introducer, ABP dropped to Zero for 2-3 s. At that time, HR was 156 bpm and EtCO_2_was 32 mmHg. After the bar was correctly positioned, ABP, HR and EtCO_2_ recovered to normal ranges spontaneously. The convexity of the previously prepared pectus bar faced posteriorly when it was passed through the track, and then, when in position, the bar was uneventfully turned over. The curvature of the anterior chest wall was assessed to be improved at the end of surgery. Temperature was 36.8 °C, when the operation was finished. The paravertebral nerve block segment was assessed by pin prick in PACU. The Face, Legs, Activity, Cry, Consolability scale (FLACC Scale) was scored at PACU and 12 h or 24 h after surgery. The patient had an uneventful post-operational recovery. Except for single shot PVBs, Diclofenac Sodium Suppositories (0.5 mg. kg-1) was given via rectum in need to relief the pain after surgery for 3 days. No pain and other complication were observed after the operation. Removal of that metal bar is scheduled for 36 months postoperative.

To identify reports for inclusion in this review, we searched PubMed for relevant studies published up to May 2017. The search was limited to studies conducted on humans. Only cases published in English were included. Search terms used included [“Poland syndrome” OR “Poland’s syndrome” OR “syndrome of Poland” OR “Poland anomaly”] and [“anesthetic management” OR “anesthesia” OR “anaesthetic management” OR “anaesthesia”].

## Discussion and conclusions

Here we report the anesthetic management of a 5-year old PS patient who underwent a Nuss procedure to correct right chest deformities related to PS. The conception of PS has been developed for nearly 180 years, since Alfred Poland reported it for the first time in 1841. Now, total or partial agenesis of the pectoralis major muscle is widely accepted as the only essential criterion for PS definition [8]. However, the original definition of PS must include the combination of TA and ULA, which was modified in 2001 in a review written by AI-Qattan, who considered the isolated form of pectoral muscle hypoplasia or aplasia as the mildest type of PS [9].

The etiopathogenesis of PS is still unknown, several hypotheses have been advanced. The prevailing theory of PS’ etiology is impediment of the embryonic blood supply at around the seventh week of gestation induced by hypoplasia of the ipsilateral subclavian artery or one of its branches with prenatal exposure to potential teratogens (cocaine, misoprostol, or smoking) that interfere with vascular development [8]. It has also been reported that less than 50% decreased size and flow velocity of the subclavian artery have been found in patients with PS [10]. This situation might lead to variable defects depending on where the restriction of blood flow occurs. Although sporadic cases of PS were reported in most articles, familial cases with different inheritance patterns have been occasionally described, indicating that PS might have a genetic basis. It could be hypothesized that the presence of different genes mutations may lead to clinical differences among subgroups of patients and the different inheritance patterns observed [11]. Array-comparative genomic hybridization (Array-CGH) analysis showed deletion (del) or duplication (dup) in different chromosomes, including11q12.3 del,6q21-q22.1 del, 1p31.1 del, Xp11.22 dup and 16q23.1 dup in 6 familial patients [9, 12, 13]; 5p15.2 dup, 5p14.3 dup, 5p14.1-p13.3 del, 6q21-q22.1 del, 7q11.22 del, 9p24.2-p24.1 dup, 11q12.3 del, 11p14.1 del, 12q21.31-q21.32 dup, 13q12.11-q12.12 del, 16p13.11-p12.3 dup or del, 16q22.3-q23.1 dup, Xp11.22 dup,14 different heterozygous chromosome anomalies in 14 different sporadic patients. These copy number variants may be involved in cell adhesion, nucleotide binding, blood coagulation, asymmetric development, chondrogenesis and skeletal muscle structure [11].

Previously reported cases were identified by searches conducted on PubMed up to May 2017 using the following key words: [“Poland syndrome” or “Poland’s syndrome” or “syndrome of Poland” or “Poland anomaly”] and [“anesthetic management” OR “anesthesia” OR “anaesthetic management” OR “anaesthesia”]. Only cases published in English were included. Four articles published between 1998 and 2014 were retrieved, and anesthetic management of 3 patients from 8-month to 23-years of age were described [14–17]. Time of publication, severity of defect, surgical procedure, pre-operational examinations, intraoperative monitoring, anaesthesia method, premedication, anaesthesia induction, airway management, anaesthesia maintenance, pain management after surgery are summarized in Table 1.

**Table 1:** Literature review of anaesthetic management of patients with PS

Title	Author/year	Age/gender	Diagnosis	Defect	Surgical procedure	Pre-operational examinations	Anaesthesia method	Premedication	Anaesthesia induction	Airway management	Anaesthesia maintenance	Intraoperative monitoring	Pain management after surgery
Anaesthesia in Poland syndrome [14].	Sethuraman R/1998	An eight-month-old boy	Left sided PS	Left upper limb hypoplasia and absence of ribs on the left side of chest wall	CT scan of the thorax/20 min	–	General anaesthesia	–	5 mg.kg^−1^ thiopentone and tracheal intubation was facilitated with 2 mg.kg^− 1^ succinylcholine.	PVC tube and the lungs ventilated manually. Ventilation was controlled with a Mapleson F system	Nitrous oxide (66%), halothane 0.5-1% and 0.2 mg.kg − 1 atracurium	Heart rate, respiratory rate, pulse oximetry, the others are not be clearly reported.	–
Letter to the editor [15].	Küpper HJ /1999	Same patient above	–	–	–	–	–	–	Children with musculo-skeletal diseases show an exceptional risk of developing succinylcholine-related complications including cardiac arrest.	–	The addition of halothane to maintain anaesthesia potentiates the risk for malignant hyperthermia	–	–
Anaesthetic Management of Patient with Poland Syndrome and Rheumatic Mitral Valve Stenosis: A Case Report [16].	Kabukcu HK /2005	A 17 year old male	PS and rheumatic mitral valve stenosis	Severe left thoracic cage deformities, with multiple abnormal left ribs and thoracic scoliosis, dextroposition of heart, severe mitral valve stenosis, grade 3 tricuspid regurgitation and severe pulmonary hypertension, congenital butonier deformity of the fingers of both hands.	Closed mitral commissurotomy/ 150 min.	The chest X-ray, arterial blood gases, the 2 dimensional and doppler echocardiographic examination, computerized tomography, respiratory function tests	General anaesthesia	0.05 μg.kg^− 1^midazolam iv	2 μg.kg^− 1^ fentanyl, 2.3 mg.kg^− 1^ propofol and 0.6 mg.kg^− 1^ rocuronium	Endotracheal intubation, intermittent positive pressure ventilation and positive end-expiratory pressure of 5 mmHg	TIVA technique using 3 mg.kg^-1.^h^− 1^propofol, 0.5 mg.kg^-1.^h^− 1^rocuronium and 10 μg.kg^-1.^h^− 1^fentanyl	Arterial pressure, heart rate and oxygen saturation, pulmonary arterial catheter was inserted after induction of anaesthesia	–
Anaesthesia in Poland syndrome: A case report [17].	Ince I /2014	A 23-year old female	Right-sided PS	A right chest wall deformity, including absence of the pectoralis major, pectoralis minor, breast and nipple, rudimentary development of 3rd rib and 2, 3, 4, 5 syndactyly of the fingers	Breast reconstruction surgery/ 4 h.	Heart and lung auscultation, respiratory function tests, echocardiography. The others are not be clearly reported.	General anaesthesia	Midazolam	2 mg.kg^−1^ propofol, 2 μg.kg^− 1^ fentanyl and 0.6 mg.kg^− 1^ rocuronium	–	TIVA by using 6 mg.kg^− 1^.h^− 1^propofol and 0.25 μg.kg^− 1^.min^− 1^remifentanil.	Oxygen saturation, heart rate and arterial pressure, body temperature and end-tidal CO_2_	–

*TIVA* Total intravenous anaesthesia, *PVC* Polyvinyl chloride

From the literature, the most common surgeries for PS, which needed anaesthesia were cosmetic surgeries such as thoracic or breast reconstruction, or diagnostic procedures like CT scan. Thoracic and breast anomalies in Poland’s syndrome without hypoplasia of the upper limb, like our case, usually had no vital functional impairment. Since these deficiencies are generally cosmetic, surgical interventions are usually performed in adulthood or late adolescence. However, in cases of severe chest deformities, the parents maybe wish for early treatment. A recent research reported patients with PS experience maximum discomfort during adolescence. Physical deformities caused by PS are prone to lead to insecurities and cause the stabilization of a body image disorder during adolescence. Moreover, if a body image disorder is stabilized, cosmetic surgery may not be sufficient and may even worsen the quality of life [18]. It is evident that early surgical procedures may have psychological benefits [19]. Surgical reconstruction during the teen years may have an extremely positive impact on a correct stabilization of body image and quality of life of young patients. When surgery is postponed to adulthood, there is no association between corrective surgery and reduction of a clinically significant body discomfort. In such situations, the surgery needs be done before adolescence, and operative trauma should be minimized to prevent any risk of growth inhibition of the thorax [20]. Our surgical team decided to use Nuss method, which is minimally invasive, as the first stage of treatment to correct sternal aberrations. If the Nuss method alone is insufficient, complete correction of developmental thoracic defects should be planned as the treatment at a later stage [21]. Such methods include muscular transfer with or without implants, reconstruction of the chest with the repair of the chest’s bone structure, and nipple-areola complex dislocation.

Standard preoperative examinations are not mentioned systematically in previous reports. Besides regular blood test, for the chest deformity, chest X-ray and computed tomography should be done to know severity of the deformity. Respiratory function tests and artery blood analysis should be done to evaluate the consequence of restriction breath and/or paradox breath. Due to the possibility of interruption of subclavian artery blood supply, bilateral subclavian artery color doppler ultrasonographyshould be done. If the subclavian artery is significantly smaller than regular size and/or low flow velocity has been reported on the impacted side, the contralateral upper limb should be used to measure NIBP or ABP. Some children with PS have heart malformation such as dextrocardia, therefore the 2-dimensional and Doppler echocardiographic examinations should be done. According to physical examination, if there are abnormalities in other organ systems in PS patients, or patients have other congenital disease, other examinations should be added. And Array-CGH analysis might reveal gene mutations in different chromosomes of PS patients.

Some experts believe that if the patient is greater than 3 years old, Nuss procedure should be performed as early as possible [22, 23], and that the method can be safely performed without a high rate of complications. However, these opinions only come from patients with pectus excavatum without any other congenital disease. Actually, achieving a satisfactory effect of correction in these PS patients with the Nuss procedure is very challenging, as reported by our case as well as others [20, 24]. There are a few factors that increase the difficulty of performing a successful correction in PS patients. First, the multiple developmental defects of the chest in PS is more complex than pectus excavatum. Second, the asymmetric character of the deformation in PS make it harder to achieve a bilateral symmetric appearance. Final, PS mostly affects right chest wall, and has a much smaller right chest cavity. However, in Nuss procedure, introducer usually advances from right chest wall, then goes through right chest cavity to left to avoid damage of heart. So smaller space is provided to surgeons as introducer advances in PS patient as compared to pectus excavatum patient. In our case, when the introducer went under the sternum from the right edge to the left, it compressed the heart massively, until the tip of the introducer got through the sternum. Under Trendleberg’s position, fluid boluses were given to ensure heart filling, and vasoactive drugs were titrated through central vein catheter to maintain satisfactory heart beat and blood pressure. During the operation, hemodynamic instability was finally overcome by multiple treatments. So besides NIBP, ABP on the unaffected side and central vein catheter are recommended for intraoperative monitoring.

For chest wall reconstruction surgery, general anaesthesia is the first choice. There is no literature that reports a PS patient suffering from malignant hyperthermia (MH), because they are both rare disorders. However, it is reasonable that patients of PS and some other congenital diseases, show an exceptional risk of developing MH due to musculoskeletal abnormalities [25, 26]. Molecular genetic studies have established chromosome 19q12-q13.2 as the primary MH locus, and chromosomes 1q32, 17q11.2-q24, 7q11.23-q21.1 or q21-q22,3q13.1 and 5p as alternative MH loci [27–32], which could share the same or similar loci of chromosome in PS as mentioned above, such as chromosomes 5p, 7q11.22. Hence, succinylcholine and inhalational agents are contraindicated in patients with PS [15]. In this current case report TIVA is used for maintenance of anaesthesia, rocuronium is used for muscle relaxation rather than succinylcholine and body temperature and EtCO_2_ were monitored because of MH risk [10].

In PS patients, a chest wall defect without underlying bone or muscle makes this condition similar to an open chest. The negative pressure generated during inspiration leads to in-drawing of the chest wall in the region of the defect and vice versa during expiration. Patients with PS can be asymptomatic and have paradoxical breathing. In our case, paradoxical breathing was absent at rest but evident with deep breathing. Uncontrolled spontaneous respiration under general anaesthesia may worsen paradoxical breathing and cause cardiopulmonary collapse. So controlled positive pressure ventilation was chosen during the procedure [17].

In addition to the possibility of paradox breath and decreased respiratory muscle strength, the lung at the side of the deformity is more likely hypoplastic or smaller. Therefore, these patients may experience a profound respiratory depression postoperatively [16]. Pain management with regional anesthesia may help to avoid additional respiratory suppression caused by intravenous analgesics. The sternum is innervated by intercostal nerves T1-T6. The incision was covered mainly by T4-T5 intercostal nerve. After risk-benefit balance information was given, the parent agreed to receive single-shot bilateral thoracic paravertebral nerve block for post operative analgesia. We selected thoracic paravertebral nerve block at the T5 level. Low concentration ropivacaine was used to preserve the intercostal muscles strength. Under ultrasound guidance, 0.25% ropivacaine 0.5 ml.kg^− 1^ with 1:200,000 epinephrine was injected on both sides at the level of the fifth thoracic vertebra in our case. After thoracic paravertebral nerve blocking, bradycardia was observed because of blocking cardiac sympathetic nerve, atropine was needed to recover the heart rate. Because the patient was given general anesthesia before the paravertebral nerve blockade, dermal block segment was measured after surgery in PACU. The block segment was from T2-T6 assessed by pin prick. The patient didn’t feel any pain until 12 h after surgery. FLACC scale is scored as “0” at PACU and 12 h after surgery and “2” at 24 h after surgery (face score 1, consolability score 1). Thoracic epidural anaesthesia can be used for Nuss procedure. Whereas, vertebral anomaly in PS patients may increase the risk of complications of epidural analgesia. Recent study reported that bilateral paravertebral nerve blocks are equal to thoracic epidurals for postoperative pain management in children undergoing Nuss procedure [33]. Single shot bilateral thoracic paravertebral nerve block with 0.25% ropivacaine with 1:200,000 epinephrine can benefit patient during the first 24 and even 48 postoperative hours following Nuss procedure in children [34]. After risk-benefit balance information was given, the parent agreed to receive single-shot bilateral thoracic paravertebral nerve block for postoperative analgesia. Adjunct drugs like clonidine or opioid may prolong the duration of analgesic effects of local anesthetics, but we don’t encourage this off label use in children for routine clinical work in China.

In conclusion, general anesthesia with controlled ventilation can be safely used for patients with Poland syndrome in chest reconstruction surgery. Examinations before surgery, intraoperative monitoring, choice of general anesthetics and pain management after surgery should all be contemplated.

## Abbreviations

Array-CGH: Array-comparative genomic hybridization
EtCO_2_: End-tidal CO_2_
MH: Malignant hyperthermia
PS: Poland syndrome
PVC: Polyvinyl chloride
TA: Thoracic anomalies
TIVA: Total intravenous anaesthesia
ULA: Upper limb anomalies
